# Supplementary material for: SARS-CoV-2 variants with mutations at the S1/S2 cleavage site are generated in vitro during propagation in TMPRSS2-deficient cells
Source: PLoS Pathog. 2021 Jan 21;17(1):e1009233. doi: 10.1371/journal.ppat.1009233 (PMC7853460; doi:10.1371/journal.ppat.1009233)
Supplement: S1 Table — The dominant sequence variants with >50% frequency were listed. Nucleotide deletions or substitutions in or near the S1/S2 cleavage motif that have been identified in this study are shown in bold. (PDF) [file ppat.1009233.s001.pdf]

S1 Table. Mutations identified in the genome of the S gene mutants

Region	Gene	Variant type	Reference*	Allele	del1	del2	del3	R685H
2482	ORF1a	Synonymous SNV	C	T	74.9			
3547	ORF1a	Synonymous SNV	A	G	77.0			
4548	ORF1a	Nonsynonymous SNV	C	T		91.8		
8183	ORF1a	Nonsynonymous SNV	T	C	77.4			
9518	ORF1a	Nonsynonymous SNV	C	A		99.8		
18093	ORF1b	Synonymous SNV	T	C	91.7			
20230	ORF1b	Nonsynonymous SNV	C	T			99.5	
22203	S	Nonsynonymous SNV	A	G			99.7	99.8
22298	S	Nonsynonymous SNV	A	C	93.1			
**23594..23623**	**S**	**Deletion**	**AATTCTCCTCGGCGGGCACGTAGTGTAGCT**	**-**	**99.4**			
**23606..23626**	**S**	**Deletion**	**CGGGCACGTAGTGTAGCTAGT**	**-**		**99.5**		
**23624..23632**	**S**	**Deletion**	**AGTCAATCC**	**-**			**99.4**	
**23613**	**S**	**Nonsynonymous SNV**	**G**	**A**				**99.7**
26963	M	Nonsynonymous SNV	T	G			99.8	99.9
27379..27381	ORF6	Nonsynonymous MNV	GAT	CTC				70.5

*Sequence of wk-521 (GenBank accession no. LC522975) is used as a reference
