# Supplementary figures and images for: SARS-CoV-2 variants with mutations at the S1/S2 cleavage site are generated in vitro during propagation in TMPRSS2-deficient cells
Source: PLoS Pathog. 2021 Jan 21;17(1):e1009233. doi: 10.1371/journal.ppat.1009233 (PMC7853460; doi:10.1371/journal.ppat.1009233)

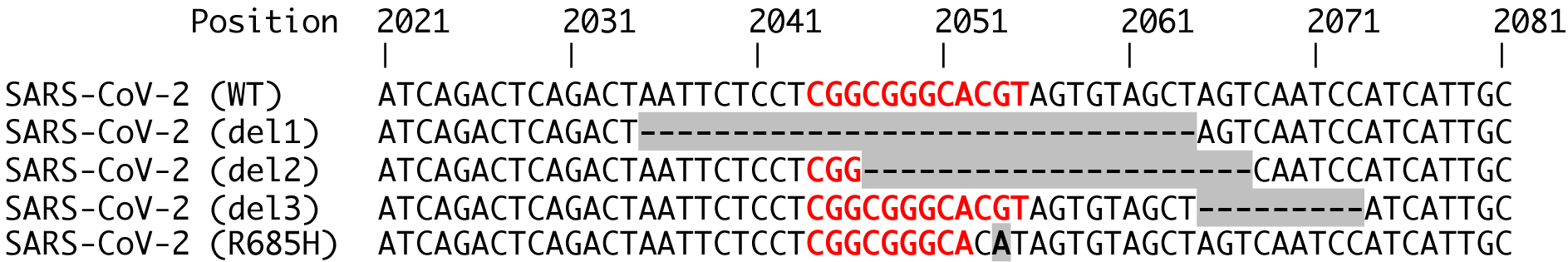

Supplement: S1 Fig — Nucleotide substitutions and deletions are shown as gray boxes. Sequence encoding the polybasic cleavage motif (RARR) at the S1/S2 cleavage site is highlighted in red. (TIF) [file ppat.1009233.s002.tif]

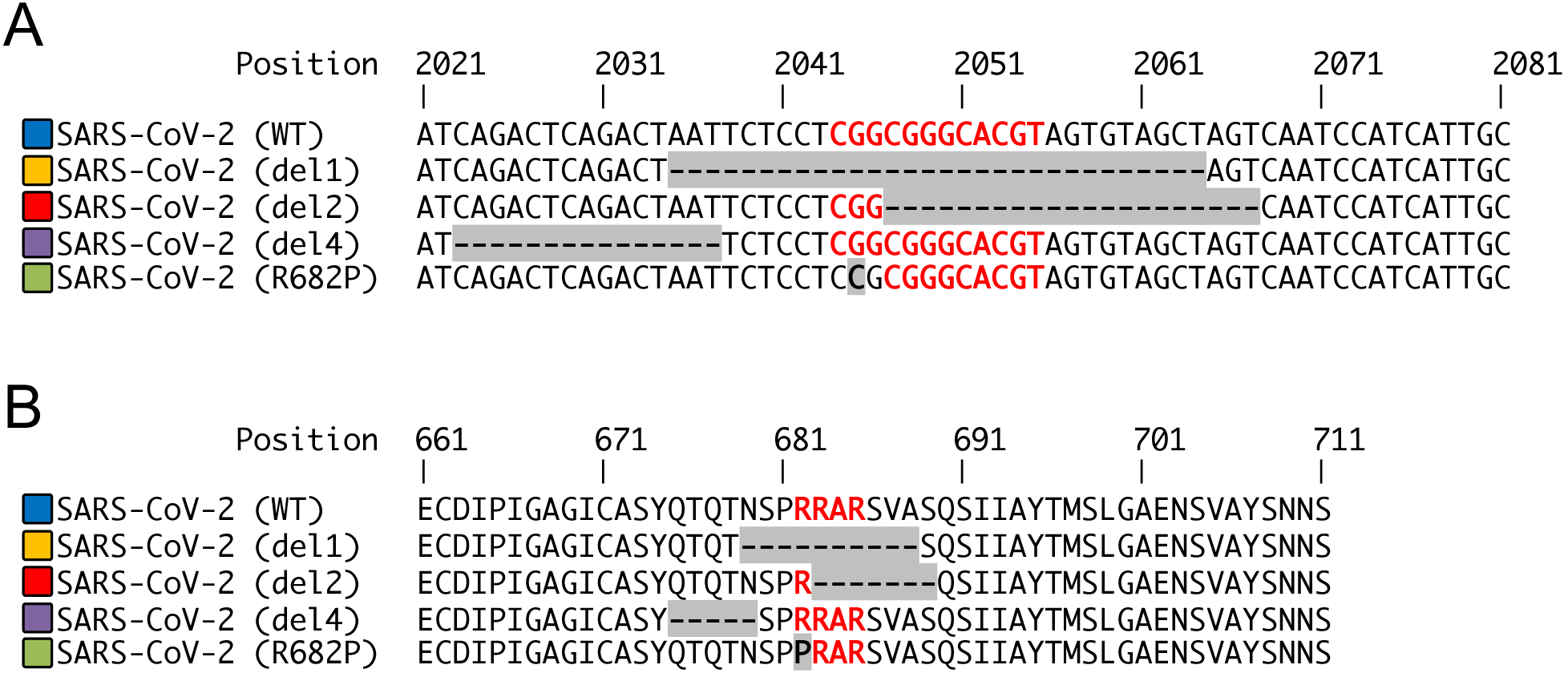

Supplement: S2 Fig — Multiple (A) nucleotide and (B) amino acid sequence alignments were constructed based on the sequence of WT and SARS-CoV-2 variants identified by deep-sequencing (related to Fig 4). Infectious viruses of del4 and R682P were not isolated in this study. Nucleotide substitutions and deletions are shown as gray boxes. Sequence encoding the polybasic cleavage motif (RARR) at the S1/S2 cleavage site is highlighted in red. (TIF) [file ppat.1009233.s003.tif]

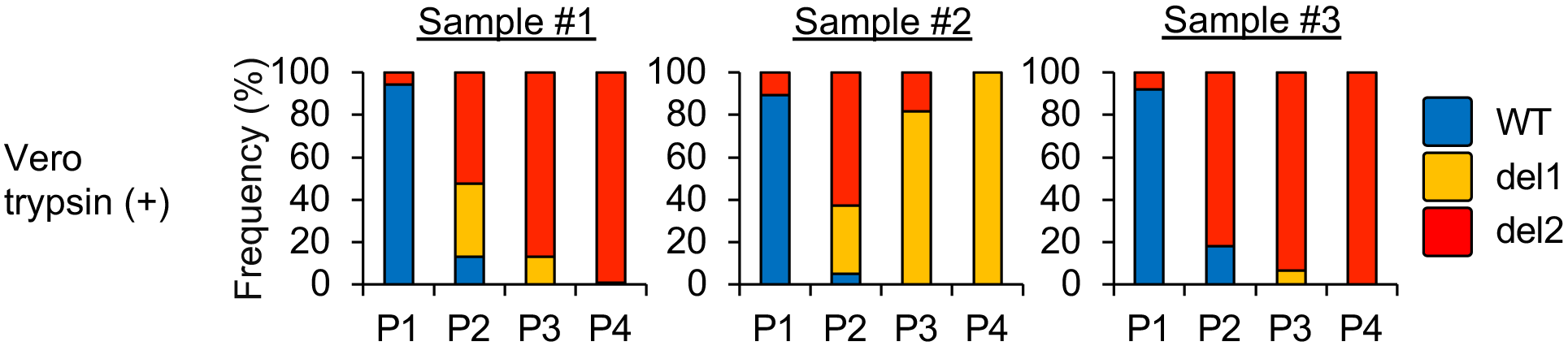

Supplement: S3 Fig — SARS-CoV-2 was serially passaged in Vero cells in serum free DMEM containing trypsin with three biological replicates. Nucleotide sequence diversity at viral S1/S2 cleavage site was determined by deep-sequencing. (TIF) [file ppat.1009233.s004.tif]
